# Parent Perspective on Care Coordination Services for Their Child with Medical Complexity

**DOI:** 10.3390/children4060045

**Published:** 2017-06-06

**Authors:** Rhonda G. Cady, John L. Belew

**Affiliations:** Gillette Children’s Specialty Healthcare, St. Paul, MN 55101, USA; jbelew@gillettechildrens.com

**Keywords:** children with medical complexity, care coordination, care management, parental burden, qualitative methods, rural, limited English proficiency

## Abstract

The overarching goal of care coordination is communication and co-management across settings. Children with medical complexity require care from multiple services and providers, and the many benefits of care coordination on health and patient experience outcomes have been documented. Despite these findings, parents still report their greatest challenge is communication gaps. When this occurs, parents assume responsibility for aggregating and sharing health information across providers and settings. A new primary-specialty care coordination partnership model for children with medical complexity works to address these challenges and bridge communication gaps. During the first year of the new partnership, parents participated in focus groups to better understand how they perceive communication and collaboration between the providers and services delivering care for their medically complex child. Our findings from these sessions reflect the current literature and highlight additional challenges of rural families, as seen from the perspective of the parents. We found that parents appreciate when professional care coordination is provided, but this is often the exception and not the norm. Additionally, parents feel that the local health system’s inability to care for their medically complex child results in unnecessary trips to urban-based specialty care. These gaps require a system-level approach to care coordination and, consequently, new paradigms for delivery are urgently needed.

## 1. Introduction

Children with medical complexity (CMC) are a subset of children and youth with special health needs (CYSHN) who have multiple chronic conditions, often require life-sustaining technology (e.g., feeding tube, central venous line, ventriculoperitoneal shunt, and tracheotomy), and/or have neurological impairment [1]. Representing less than 1% of U.S. [2] and Canadian [1] children, this subset require greater resources than most CYSHN to optimize their functioning and health status [3,4], and rely on care from multiple primary, specialty, and service providers. Communication and collaboration between providers and services is therefore essential [5], and the need for coordinated care has been well-documented [6,7].

Patient- and family-centered care is described as “… an approach to the planning, delivery, and evaluation of healthcare that is grounded in mutually beneficial partnerships among healthcare providers, patients, and families” [8]. The recommended approach of primary care delivery for all persons, including children with medical complexity, is the patient- and family-centered medical home model (FCMH) [6,9]. Coordination of care is an essential component of the model [10,11] and encompasses three overarching components: (1) placing the child/family at the center of the process, with involvement in all decisions in order to promote self-care and advocacy; (2) developing a proactive, comprehensive, current, and accessible plan of care; and (3) utilizing teamwork, collaboration, and communication across settings [5,7]. In the FCMH model, locus of care coordination typically resides with primary care [12] and a key facilitator of effectiveness is communication and co-management across settings and persons [6]. A summary of evidence-based care coordination functions are listed in Table 1.

Implementation of the FCMH model in high-performing, tertiary-based programs providing primary and specialty care for CMC found increased family satisfaction with overall care [13,14,15], increased quality of life [16], reduced parental burden [17,18], reduced unmet health service needs [19], decreased unplanned or emergency admissions [20], and in some cases, reduced health services cost [21,22,23]. Despite recommendations and implementation success, data from the 2011–2012 National Survey of Children’s Health reveal that less than half of CYSHN receive care in a medical home [24]; for children with medical complexity, the numbers are even smaller [25]. Investigation into why the most vulnerable children are not receiving a standard of care designed to improve their health and well-being reveals ongoing barriers from both provider and parent perspectives.

Continuity of care is defined by three dimensions. Informational continuity ensures prior health information is used to inform current health decisions and care; management continuity utilizes shared plans of care across systems to ensure consistent and coordinated services; and relational continuity involves ongoing relationships with consistent healthcare personnel [26]. Families often report that their greatest challenge is fragmented communication across systems, services, and providers [17,18,27,28,29] along with informational and management continuity gaps, both within and across systems of care [30,31]. When their child lacks a designated “in-charge” person, parents often assume the role of primary care coordinator [17,18], aggregating and sharing health information from multiple providers [32]. While parents could often serve as the primary communicator and source of information between systems of care, they lack electronic and informational tools to properly facilitate this role [33].

Underpinning these findings is the gap between family-centered care conceptualization and translation. Widespread agreement exists on the three domains of family-centered care: “(1) valuing parents’ knowledge and experience; (2) supporting parents in their role as care giver; and (3) incorporating parents’ expertise into clinical and psychosocial care” [34] (p. 146), but poor implementation into clinical practice still persists [34]. Providers and family caregivers describe gaps in hospital-to-home transitions for CMC that include effective engagement and communication with families and care coordination to ensure discharge readiness [35]. Many practicing pediatricians view FCMH implementation to be vital for improving child health and resource use, yet over half describe lacking the personnel resources to facilitate the activities [36]. Finally, a lack of community-based primary care providers, especially in rural areas [37,38], limits access to the medical home for CMC.

In 2008, the state of Minnesota passed health reform legislation, laying the foundation for the development and implementation of a voluntary program for state certified medical homes [39]. Grounded in primary care, a fundamental component of the state certification program is improved coordination and communication between clinicians and services [40]. The Minnesota Department of Health’s Children and Youth with Special Health Needs Strategic Plan 2013–2018 [41] guides statewide development, coordination, and improvement of the system of care for CYSHN. A key objective of the Strategic Plan is to increase access to coordinated, comprehensive care by building the capacity of certified medical homes to work with CYSHN [41]. To help achieve this objective, a three-year program was funded, testing a primary care-specialty care (Primary‑Specialty Care Coordination Partnership for Children with Medical Complexity; PRoSPer) [39] partnership model of care coordination for children with medical complexity.

PRoSPer’s overall objective is clinical implementation and program evaluation of a specialty care-based care coordination team that partners with a child’s primary care clinic team, in order to increase communication and collaboration and provide comprehensive, family-centered care coordination. The PRoSPer specialty care coordination team resides at a tertiary urban specialty health system caring for medically complex children and adults with child-onset disabling conditions, including cerebral palsy, spina bifida, muscular dystrophies, epilepsy, and congenital syndromes. The specialty health system includes a 60-bed acute care hospital located in a large urban area, six outpatient specialty clinics in the same urban area, and 14 additional specialty clinics located in rural areas throughout the state.

A requirement of PRoSPer funding is to serve families of CMC living in rural communities or newly emigrated and non-English speaking. An agreement on PRoSPer model implementation with four primary care clinics (A, B, C and D) which met these criteria was obtained. Clinic A provides primary care to non-English speaking, newly-emigrated families from Southeast Asia, and is located in the same urban community as the specialty health system. All interactions with families from this clinic include an interpreter. Clinics B, C, and D are located in rural areas of the state, and the distance from each primary care clinic to the urban specialty health system is 90, 200, and 230 miles, respectively. Clinics A, B, and D, are statecertified medical homes, and Clinic C is pursuing certification.

**Figure 1:**
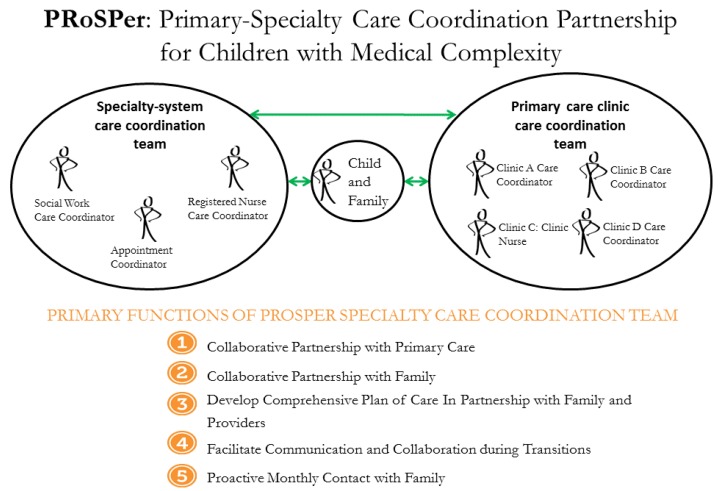
A visual representation of the Primary-Specialty Care Coordination Partnership for Children with Medical Complexity (PRoSPer) model.

The PRoSPer model is designed to bridge the communication gap between specialty and primary care (Figure 1). The specialty care-based care coordination team includes a dedicated Registered Nurse (RN), a Social Worker (SW), and an Appointment Coordinator. The Appointment Coordinator is unique to and a vital component of a specialty-based care coordination team. Since the specialty health system has over 300 providers that see patients across 18 different locations, scheduling specialty appointments in a manner that meets the family’s needs is a primary goal of the project. The primary activities of the RN and SW specialty-based coordinators include: meeting with families during specialty clinic appointments to assess needs, developing a comprehensive plan of care that is shared with families and the primary care provider, and communicating and collaborating with primary care after clinic visits and hospitalizations (primarily by telephone, fax, and secure email; Figure 1).

The PRoSPer specialty-based care coordination team worked with each primary care clinic to identify approximately 10 children eligible for participation in PRoSPer. The children identified were medically complex and under the care of three or more specialists from the specialty health system. Parents of identified CMC were contacted by the PRoSPer specialty-based care coordination team and offered participation in the new clinical program. All parents (*n* = 38) expressed interest in receiving PRoSPer care coordination and from this group of 38 families, 30 were recruited to participate in a separate program evaluation.

This small study is a component of a PRoSPer program evaluation, and parental focus groups were conducted during the first year of PRoSPer implementation. Focus group methodology was chosen over individual interviews to facilitate and support parental sharing of care coordination perceptions and experiences [42]. The aim of this qualitative study was to understand how the parents of medically complex children enrolled in PRoSPer perceive communication and collaboration between the providers and services delivering care for their child.

## 2. Methods

### 2.1. Setting and Subjects

The study was conducted in accordance with the Declaration of Helsinki, and the protocol was approved by the Institutional Review Board of the University of Minnesota (1412M57201). From the group of 38 families receiving the PRoSPer model of care coordination, 30 provided written informed consent to participate in a formal program evaluation (approximately 5–8 children from each of the four primary care clinics). An interpreter facilitated the informed consent process for non-English speaking participants.

### 2.2. Data Collection

Data were collected during four focus group sessions held in the participant’s community. Participants in each focus group were homogenous in terms of primary care clinic and geographic location. The composition and number of focus groups followed data collection recommendations to ensure the emergence of themes between and across the groups, with a recommended number of 5–7 focus group participants [42]. The Appointment Coordinator delivered an invitation for the focus group session four weeks in advance, via telephone, to the 30 eligible families enrolled in the PRoSPer evaluation (5–8 invitations per focus group).

**Figure A1:**
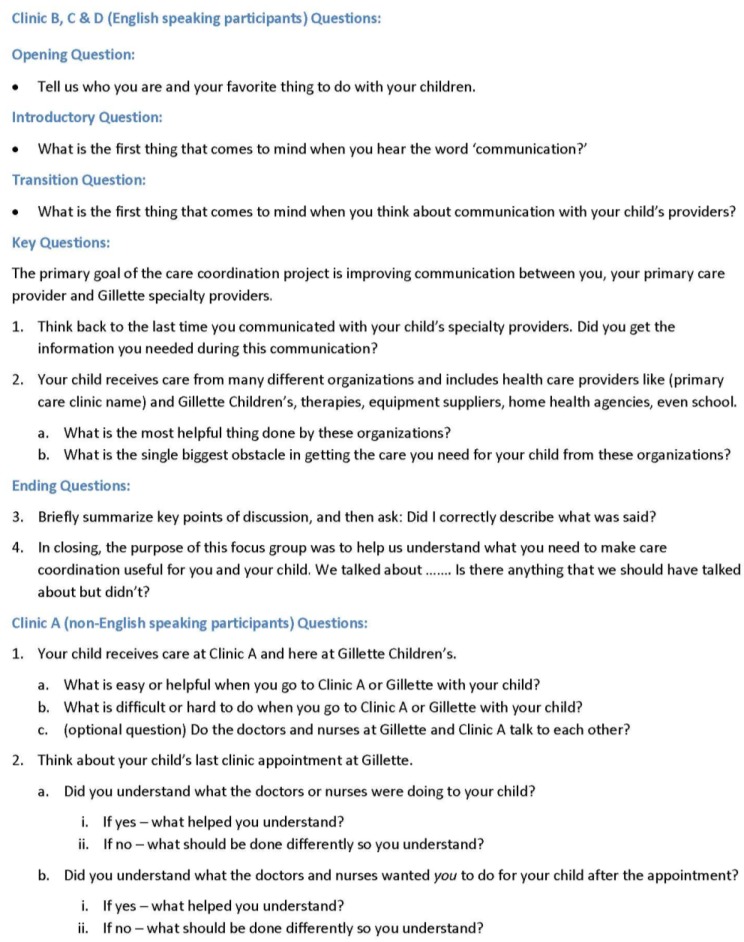
Parent focus group questions.

Each focus group session lasted 60–90 min and took place in a private room [42]. Focus group questions were developed following the guidelines of Krueger and Casey [42] and included opening, introduction, transition, key, and closing questions (Figure A1). For the session with Clinic A (non‑English speaking participants), questions were modified to include concrete statements asking about the activities of care coordination, instead of the abstract concept of care coordination. All focus groups were led by an experienced interviewer and included one or two members of the care coordination team for note-taking. This protocol was the same across all clinics, with the exception of Clinic A, which included an interpreter. All focus groups were audio-recorded for later transcription.

### 2.3. Data Analysis

Conventional content analysis [43] of the focus group transcripts used an iterative and inductive process, reading and coding transcripts to identify emergent themes [44] and describing the phenomenon of parental perception of communication and collaboration between the providers and services providing care for their child. All audio-recordings were transcribed verbatim. No a priori themes were utilized. The content analysis was conducted by the authors using NVivo 10 for Windows, Version 10.0.641.0 SP6 (QSR International, Doncaster, Victoria Australia).

Rigor (internal validity, reliability, and external validity) was evaluated using the naturalistic inquiry criteria of Lincoln and Guba [45] via member checking and secondary coder review. Member checking of findings occurred with an unrelated group of seven urban/suburban English-speaking parents of children with medical complexity, instead of the original focus group participants, and was selected to confirm that emergent themes represent the perspectives of a broader sample of parents [46]. Secondary coder review was performed by six members of the specialty organization Nursing Research Committee (NRC). Agreement in coding ranged from 72% to 88% (mean = 79%). All disagreements were reviewed by the primary coder and 25% (*n* = 9) resulted in recoding of the transcript data.

## 3. Results

The unpredictable nature of medically complex children and the demands of work and family resulted in only two parent participants at each of the four focus group sessions. The eight parents (five mothers and three fathers) represented seven children. Seven of the parents were ‘unpaid’ caregivers for their child, while one parent had a state-specific waiver that compensated the parent for providing personal care attendant level care for their child. Demographics of participants and their children are shown in Table 2.

Final themes emerging from the focus group data fall into four broad categories: care coordination delivery, communication and collaboration, access to care and services, and family impact. In the context of this study, the overarching theme expressed by parents was learning how to care for and manage their child’s complex medical conditions, and parents assuming the role of 24/7 care coordinator. Parents described these roles evolving over time and referred to this as “figuring it out on my own.” Additionally, several parents described how they learned care coordination skills from other parents. Web-based resources and social media were also useful, although parents acknowledged the potential for misinformation from informal sources.

### 3.1. Care Coordination Delivery

We found that care coordination is a dynamic process that takes into account the complexities of both child and family life. Parents did not perceive care coordination as a cohesive responsibility or role; instead they recognized episodes when their child’s care was coordinated or “in sync,” and episodes when the care was clearly unsynchronized. Parents described the importance of finding “the right channels” and hoping for some sort of “road map” to help them navigate the complexities ahead.

- “The wheels are always turning, thinking, you know: What do we need? What’s next? How do we get this? How do we make it better, faster? How does it work better for us and [child]?”
- “If somebody would walk me through all that mess that would be nice.”
- “It’s about proper care and easiest access to it.”
- One parent described how a specialty doctor routinely reviews the child’s upcoming visits with other specialists; “he’s making sure nothing’s getting missed.”

Parents also appreciated the contributions of professional care coordinators, but were unsure of the depth and breadth of care coordination services:

“What is your role? I mean are you going to do just the tasks that a normal parent with normal kids would need done, or are you going to do all the legwork for me? If I have an issue, how far are you going to take that issue? Do I have to constantly follow up with you, weekly, to see where you’re at with it vs. where I could have just made the phone call myself and did it myself?”

The functions of care coordination delivered by their child’s primary care clinic also varied. Only two of the clinics participating in PRoSPer are well-established pediatric medical homes with dedicated care coordinators. Parents from these clinics described how to use these personnel to positively impact their child’s care.

- “When I had to have some medical forms and a couple of things faxed to the school, I talked to N (primary-care based care coordinator), and I dropped my child off at the school and they’re like, ‘Oh I got those forms, but I had a question about them (the forms)’ and I’m like, ‘Whoa. Awesome.’”
- “The minute you find something, like M (child) had a drug that I will never let her have again, some type of anesthesia, I had N put it in every place she could put it.”
- “He’s helping me by interpreting sometimes, and helping my son to get an appointment or something like that. And he also helps me with medication … he will call the pharmacy to refill my meds.”

However, parents from primary care clinics that are not well-established pediatric medical homes expressed a different viewpoint.

- “Yeah, it (primary care record) doesn’t have any of her recent breaks or doesn’t have the fact that she has a trach … or any of her information.”
- “I’m like, ‘isn’t there someone up here that is our go-to?’ I know you guys are trying, but you’re so far away. You don’t know the people, the staff, the teachers, some of the doctors in the area.”

Although parents expressed obstacles to caring for their child with medical complexity, they also described facilitators, aligning closely with evidence-based care coordination functions, which support them in caring for their child. Equally important are parent-to-parent support networks, which participants described as “finding on their own” via social networking. A summary of quotes describing care coordination functions perceived as useful when present, and the subsequent challenges when these functions are absent, are listed in Table A1.

### 3.2. Communication and Collaboration

The state where this study took place has one major urban area, and the majority of pediatric specialists are located in three different health systems within this urban area. A fundamental goal of the FCMH and evidence-based care coordination is communication and collaboration between the parent, provider, and other health system professionals, which is facilitated by a “professional” or health system-assigned care coordinator. Regardless of the primary care system, all parents expressed frustration working with the multiple systems and the lack of communication and coordination between them.

Communication and collaboration gaps are compounded by having care coordinators that are “tied” to a specific health system. Parents expressed uncertainty if one system’s care coordinator could help with issues arising in another system. One parent illustrated this frustration by stating, “*Do I have to coordinate the care coordinators too*?” Other responses in this area included:

- “I think they (systems treating child) all have the same goal in mind, but what I think would be even better is for all of them to communicate with each other."
- “You have to tell your story over and over… with the new doctors all the time. It should be there.”
- “XX (from ‘Y’ specialty system) calls me every month, and I’ll tell her, ‘yep, everything’s good.’ But I have a ton of stuff going on with W (other specialty system) that does not involve Y, that I’m drowning with over there trying to learn and figure out, you know, and wondering can XX help me?”

The lack of across system communication and coordination ultimately results in parents serving as their child’s 24/7 care coordinator. While parents report willingly assuming this role, they are frustrated by the lack of resources to meet these expectations and responsibilities. A frequently described resource that is often missing is accessible and consolidated information about their child’s condition and past health history, which results in the corresponding fear of forgetting to communicate important information.

- “I mean even if we just had their chart completely right in front of us and they… I could hand that to Dr. X and he could look: meds, history, fractures, with just little tabs.”
- “Say that the parent failed to mention about something because we’ve got 10 doctors. I can’t remember all the surgeries my kid’s had, how many times she’s been under anesthesia. I’m just like, ‘The notes are there. Read them.’ I don’t know. I forget stuff; I can’t remember.”

Poor coordination of information is aggravating, but also poses a significant risk for harm to the patient. Parents expressed the need for constant vigilance to ensure vital information wasn`t missed. One parent described frustration with the multiple health systems’ inability to keep track of medications: “*Every time that you go down there you (go) through meds, and then you come back up here and you go through meds, and then you come back the next time and you go through meds and they’re still wrong.*”

### 3.3. Access to Care and Services

Three of the primary care clinics participating in PRoSPer reside in rural areas of the state. A commonly expressed concern was the inability of the local health system to care for their medically complex child in urgent and emergent situations, especially when technology (e.g., feeding tube, tracheostomy) is present. Primary care providers are trusted members of the care team, but may have limited expertise in managing the child’s complex needs. Several parents described frequent transport to a specialty tertiary center hundreds of miles away. Parents understood that local providers make this decision in the best interests of the child and to facilitate access to needed care, but explained corresponding financial and emotional impact on their family.

Another major obstacle expressed by parents is accessing, using, and maintaining social service resources. Public health, social, and waiver services are managed at the county level, and moving residence across county lines means not only reapplying for essential services, but losing trusted public health nurses and county case managers. Although a child is eligible for services in the prior county, the new county often requires another eligibility assessment and application, with a corresponding gap in needed services. Another obstacle is the need for the yearly renewal of insurance and social services. Parents expressed frustration with this requirement since their child will never “get better.” Newly-immigrated parents described additional obstacles of language and cultural differences, and immigration from extremely rural locations or refugee camps.

### 3.4. Family Impact

Parents consider 24/7 management of their child’s care a top priority, but illustrated how this role has significant impact on their family life. Parents describe having little time for other children, their spouse, and more importantly, themselves. Jealousy of the “extra attention” given to the child with medical complexity was commonly stated; siblings want mom and dad to themselves and are frustrated when their sibling’s condition prevents or interrupts a family outing. Additionally, parents expressed worry about placing too much responsibility on siblings.

## 4. Discussion

At a system and organizational level, healthcare strives to ensure high quality, cost-effective care. For persons of all ages with complex chronic conditions, care management and care coordination have been identified as mechanisms to achieve this goal [7,47]. Research and quality initiatives for CMC and their families have focused on developing programs that meet these goals, by bridging coordination gaps within and across systems of care.

In this study, we explored how parents of medically complex children participating in the PRoSPer program perceive communication and collaboration between the providers and services caring for their child. Focus group data from parents of medically complex children that are newly emigrated and non-English speaking or living in rural areas perceived receipt of evidence-based care coordination (Table 1) as a key facilitator of communication and collaboration between the providers and services caring for their child. Parents are appreciative when these care coordination functions occur, but receipt is often the exception and not the norm. Parents reported that communication across systems of care can be fragmented and uncoordinated, and this gap in communication is often perceived as a threat to their child’s health and well-being. Many parents try to eliminate this gap by assuming responsibility for their child’s 24/7 care and care coordination, however, they often encounter numerous obstacles and barriers to fulfilling this role, but describe situations where delivery of evidence-based care coordination reduces these obstacles and benefits their child.

Many of the obstacles described by the PRoSPer focus group participants have been previously documented and underscore that ongoing challenges of parents caring for a medically complex child have not yet been adequately addressed. Similar to the participants in our study, parents develop expertise in caring for their child’s complex needs, but often have difficulty obtaining needed information or guidance from health professionals [48]. In addition, parents provide a significant amount of unpaid care for their children [49,50]. Like our participants, other parents of children with medical complexity revealed the impact of the 24/7 parental care and care coordinator roles on family life, with reduced time for relationships with other children and spouses [17], and that these roles dominate their role as a parent [29]. Despite the disruption of family life, many parents work to maintain a normal family environment, and often engage with parent support networks, which can provide needed support [48].

Our findings reflect the continued concern over barriers to delivering evidence-based care coordination at the system level, which has also been described in prior studies [17,18]. Barriers to information sharing that were expressed by parents include a fragmented health system with poorly-defined care coordination role accountability, lack of cross-systems comprehensive and accessible health information, and lack of standardized policies and processes for sharing information [29]. Recommended strategies to reduce these barriers include inter-agency communication and coordination and a “system-wide information management solution” [18]. Electronic sharing of data across systems is a goal of the HITECH Act [51] and state-implemented health information exchanges, but the realization of this goal is yet unachieved, resulting in the continued industry standard of faxing or mailing information that cannot easily integrate into the electronic health record (EHR). As described by parents in this study, the primary “solution” for fragmented cross-systems communication and coordination has become the responsibility of the parents of children with medical complexity, even though they often do not have tools and information to effectively fulfill this role.

Co-management is emerging as a model to bridge communication and coordination gaps [52], with the locus of care residing mostly with specialists who partner with primary care providers. A survey of New Hampshire pediatricians and family practitioners found strong agreement with a co‑management model, especially for infrequently-seen conditions [53]. Investigation regarding the impact of co-management models has found improved communication [54] and decreased inpatient utilization [16,55,56]. As expressed by our parent participant’s, families want to stay close to their community. Increased collaboration and co-management between specialty and local providers is needed to empower local primary care and emergency department providers to care for medically-complex children in their community.

Subsequently, increased collaboration and role accountability between primary care and specialty care-based care coordinators is needed to ensure that critical health information does not ‘fall through the cracks’ because a parent, often juggling work, family, and other children, simply forgets or is not told to communicate critical health information. While EHR-supported patient portals allow parents access to their child’s medical record, the information ‘pushed’ to the patient portal reflects only care received from the specific health system. CMC often receive care from multiple health systems and parents must access multiple patient portals to obtain a full picture of their child’s health information. 

It is important to note that this study does have limitations that impact the validity and generalizability of its findings. Focus group participation at each of the four primary care clinics was low, with only two parents per group, and therefore can only provide a limited viewpoint on the perception of care coordination services and parental experiences. Funding for PRoSPer model implementation required that families of enrolled CMC reside in a single state, and live in rural areas or are newly immigrated and non-English speaking. Only two of the seven enrolled CMC had multiple, prolonged hospitalizations in tertiary settings. Our participants’ experiences and viewpoints may also not reflect parents of CMC living in urban/suburban locations, other areas of the country, or those who experience high tertiary center utilization. Additionally, members of the specialty-based care coordination team were present at the focus groups to assist with note-taking, and their presence could have influenced participant responses, although the parents’ reports of both positive and negative examples of primary care and specialty care communication and collaboration suggests minimal influence.

## 5. Conclusions

A “system-wide information management solution” [18] for electronic sharing of comprehensive health data remains elusive, necessitating a new paradigm of care coordination delivery for CMC. Parents are still having to be responsible as the primary “keepers” of their child’s health information and provide an invaluable service sharing this information across systems of care. Cloud-based, personal health records (PHRs) that meet privacy standards and regulations are re-emerging as a mechanism for consolidating health information in a single accessible location that supports access anytime, anywhere, while maintaining control over who sees this critical information. The primary disadvantage of PHRs is the need for manual updating by parents, who subsequently continue to assume the role of 24/7 care coordinator. 

Systems providing care and care coordination must work with parents to delineate roles, responsibilities, and accountability. The implementation and testing of novel models of care coordination that help parents understand the role of care coordinator and teach skills to manage their child’s care is crucial to ensure effective utilization of not only societal resources, but parental and family resources as well.

## Figures and Tables

**Table 1:** Evidence-based functions of care coordination [5,7].

Establish a relationship and patient/family expectations with dedicated care coordination visits Facilitate on-going communication between families and professionals Provide a “single point of contact” for family, providers, and community resources Assess child and family needs (psycho-social and health-related) Develop a written plan of care with the family that integrates information from multiple sources/providers and includes patient- and family-centered goals Coordinate and track referrals, test results, and treatment outcomes Facilitate access to needed care Educate families on care needed for child’s specific condition Coach family’s advocacy skills to access related educational and social support resources Support care transitions (practice to practice, pediatric to adult) Facilitate family-centered team meetings, including external organizations, when needed Use health information technology to deliver, monitor, and evaluate care coordination effectiveness

**Table 2:** Demographics of focus group participants (*n* = 8) and their children (*n* = 7).

**Focus Group Participants (Parents; *n* = 8)**	
**Relationship to child**	
Mother	5
Father	3
**Primary spoken language**	
English	6
Karen	2
**Geographic location of residence**	
Rural	6
Urban	2
**Children of Focus Group Participants (*n* = 7)**	
**Age**	
0–4	2
5–10	4
11–15	1
**Gender**	
Female	5
Male	2
**Primary underlying condition**	
Cerebral Palsy	3
Osteogenesis Imperfecta	1
Chromosomal Abnormality	1
Sacral Agenesis/Spina Bifida	2
**Technology dependent**	
Feeding tube	3
Tracheostomy	1
**Neurological impairment**	
Yes	6

## Appendix A

**Table A1:** Parental perception of the presence of evidence-based care coordination functions.

Evidence-Based Care Coordination Function	Present or Absent	Quote
Establish relationship and expectations with dedicated care coordination visits.	Present →	*“She (doctor) would always say, ‘You’re the mom’ She just had a keen sense of knowing I wasn’t just calling her because I was making this up. I was really calling her because I needed something, and she trusted me and that is a big deal.”*
		*“I’ve been fortunate enough that the last couple of times (in emergency department (ED)), both doctors are like, ‘What do you think? You know her best. Does she need hydration or does she need…?’ You know, I’m okay with them asking me those types of questions and I feel comfortable saying if she does need that.”*
	Absent →	*“What is your role? I mean are you going to do just the tasks that a normal parent with normal kids would need done, or are you going to do all the legwork for me? If I have an issue, how far are you going to take that issue? Do I have to constantly follow up with you, weekly, to see where you’re at with it vs. where I could have just made the phone call myself and did it myself?”*
Facilitate on-going communication between families and professionals.	Present →	*“The minute you find something, like M had a drug that I will never let her have again, some type of anesthesia, I had N (primary-care based care coordinator) put it in every place she could put it.”*
	Absent →	*“I think they (systems treating child) all have the same goal in mind, but what I think would be even better is for all of them to communicate with each other.”*
Provide a “single point of contact” for family, providers and community resources.	Present →	*“And I can leave this message for L, and she might be a day out, but I know she’s going to get back to me. So there’s this level between them.”* *“It’s nice, it’s super helpful, to have somebody that you know you can call.”*
	Absent →	*“I’m like, ‘Isn’t there someone up here that is our go-to?’ I know you guys are trying, but you’re so far away.”*
Assess child and family needs (psycho-social and health‑related).	Present →	*“Every time I go to the appointment, the nurse or the doctor, or whoever, working here helps me to call the ride for the return and they help me with everything. Yeah, if no one was helping me, then I would be lost in the building.”*
Develop a written plan of care with family that integrates information from multiple providers and includes patient- and family-centered goals.	Absent →	*“No one ever asked me, ‘We should get your notes from your occupational and physical therapists to see what her current needs are and where they’re sitting at,’ to actually look into my daughter’s history.”*
Coordinate and track referrals, test results and treatment outcomes.	Present →	*“I think it’s been a lot easier since we moved everything over to X. I mean, there is just so many more, so many other little things at W that were kind of difficult.”*
	Absent →	*“Yeah, it (primary care record) doesn’t have any of her recent breaks or doesn’t have the fact that she has a trach on… or any of her information.”*
Facilitate access to needed care.	Present →	*“I think it’s been a lot easier since we moved everything over to X. I mean, there is just so many more, so many other little things at W that were kind of difficult.”*
	Absent →	*“She was dehydrated. Oh, she had some sort of infection. If they just could have figured that out and got an IV in her then we could have stayed up here … and not went down to specialty hospital for a weekend with a helicopter ride.”*
Educate families on care needed for child’s specific condition.	Absent →	*“So emergencies, I just figure we’ll rely on ourselves and get to (local ED) so (local ED) can call (specialty health system) and they can say, ‘okay, bring her down.’”* *“Kind of figure it out for myself.”*
Coach family’s advocacy skills to access related educational and social support resources.	(not discussed)	
Support care transitions (practice to practice, pediatric to adult).	Absent →	*“And it could be something like they didn’t want to do a catheterized urine sample. I’m like, ‘It has to be. It’s per her specialty urology orders,’ but you go to urgent care and you just don’t know how. And literally, my husband and I did the sample. We’re like, ‘Get us a catheter and we’ll do it.’”* *“So what happened in the miscommunication, you could only have Botox three months apart. M got her Botox in May by Dr. X, in her bladder, which meant she had to wait another three months to get any Botox (in her legs), period.”*
Facilitate family-centered team meetings, including external organizations, when needed.	(not discussed)	
Use health information technology to deliver, monitor and evaluate care coordination effectiveness.	Absent →	*“I think in an ideal situation, they would give something to the parents that had everything electronically right there. Wouldn’t that be amazing?”*
